# Producing Enhanced Yield and Nutritional Pigmentation in Lollo Rosso Through Manipulating the Irradiance, Duration, and Periodicity of LEDs in the Visible Region of Light

**DOI:** 10.3389/fpls.2020.598082

**Published:** 2020-12-18

**Authors:** Laura Cammarisano, Iain S. Donnison, Paul R. H. Robson

**Affiliations:** ^1^Institute of Biological, Environmental and Rural Sciences (IBERS), Aberystwyth University, Aberystwyth, United Kingdom; ^2^Next-Generation Horticultural Systems, Leibniz-Institute of Vegetable and Ornamental Crops (IGZ), Grossbeeren, Germany

**Keywords:** LED, blue light, anthocyanin, red light, diel cycle, red lettuce

## Abstract

Pigmented food are an important part of the human diet, and anthocyanins have demonstrable protection against tumor production in mouse models and beneficial effects on human liver chemistry. As such, producing pigmented crops is important for a nutritionally diverse diet. Lollo rosso lettuce is a fast-growing pigmented plant, is rich in phenolic compounds, and represents a suitable system to test optimization strategies for yield and anthocyanin production. High-energy UV wavebands are often used to stimulate increased pigmentation; however, we hypothesized that optimizing visible wavebands would deliver both yield and quality improvements. Growing Lollo rosso under irradiances between 5 and 180 W m^–2^ using visible waveband LEDs produced 0.4 g fresh weight per W m^–2^ in the linear portion of the curve between 5 and 40 W m^–2^ and achieved an approximate asymptote of 20 g fresh weight at around 100–120 W m^–2^ for yield. Anthocyanin content increased linearly with irradiance. We attempted to optimize the visible wavebands by supplementing half the asymptotic energy for 15 days with supplemental red (R) or blue (B) wavebands in the peaks of photosynthetic activity (430–460 and 630–660 nm). R and B affected rosette morphology with no significant impact on yield, but B significantly increased anthocyanin content by 94% compared to R. We therefore focused on further optimizing B by shortening the daily duration of supplemental B. The minimum B treatment that lacked significant pigment induction was 1 h. We hypothesized that short durations would be more active at different times in the diurnal cycle. Supplemental B was applied for 2 h at four different times. A night-break with B produced the highest yield and anthocyanin content. Our research demonstrates new ways to efficiently use readily available LEDs within the PAR wavebands to increase both yield and crop quality in controlled environment agriculture.

## Introduction

It is expected that available land per capita to cultivate food will decline due to population growth and climate change. Possible alternatives to increase land use efficiency include using efficient closed-environment agriculture which can produce more crop per unit area (Touliatos et al., 2016). A revolution in lighting allows such systems to utilize efficient LEDs which allow potential control over both irradiance and spectra and, when used in completely enclosed environments, photoperiod (Bantis et al., 2018). Lighting represents a major cost, and therefore, it is important to understand how the increased flexibility achievable in new LED lighting may be best utilized to maximize returns from both crop yield and quality.

Increasing the photon flux density (PFD) results in a linear increase in photosynthetic rate (Kelly et al., 2020) until light exceeds photosynthetic capacity and photosynthesis becomes limited by other factors such as CO_2_ (Herron and Mauzerall, 1972; Robinson, 2001). Light use efficiency may also be limited by photoprotective strategies that reduce the risk of photo-oxidative damage by limiting the light interception and absorption and by enhancing dissipative routes (Adams and Demmig-Adams, 1992; Takahashi and Badger, 2011). Such strategies evolved in highly variable environments, and plants can therefore generally tolerate a range of light intensities (Ruban, 2009). Indoor cultivation, where artificial lamps are the sole source of illumination, reduces variability and allows growers to adopt optimal irradiance levels for yield, morphology, and energy costs (Kozai, 2013). Growing plants under optimized light conditions can lead to high yield and high-quality produce (Ouzounis et al., 2015). The exact light recipe may require a balance of light that is optimally used for photosynthesis and that also induces other characteristics of commercial value such as pigmentation and morphological changes that under natural conditions are responses to light stress.

Growing plants under monochromatic R or B appears to be unsuitable for most plants tested so far; monochromatic B results in a decline in photosynthetic activity (Lichtenthaler et al., 1980) and monochromatic R in the deleterious red light syndrome (Matsuda et al., 2004). A combination of R and B radiation, with notable variations in the importance of the ratio between R and B, represents a suitable and efficient light spectrum for crop growth and development (Kim et al., 2004; Izzo et al., 2019). However, a broader spectrum may be more beneficial for crop growth (Pennisi et al., 2019) and addition of green light to the growth spectrum enhances plant growth and development (Johkan et al., 2012) and increases light to the lower plant canopy (Terashima et al., 2009). Replacing a part of the broad-spectrum background with an appropriate fraction of B or R light may increase biomass accumulation (Kaiser et al., 2019), produce regular crop morphological and physiological characteristics, and enhance crop quality (Li and Kubota, 2009).

The types of crops predominantly grown in controlled environments under LEDs have tended to be limited to leafy greens and micro herbs (Benke and Tomkins, 2017). Many studies have focused on lettuce as an important global crop that responds well to light treatment (Son and Oh, 2013). Lollo rosso lettuce is rapid growing and red pigmented, the major pigment being the anthocyanidin cyanidin (Ferreres et al., 1997). Anthocyanin showed anti-carcinogenic activity in cell culture models and in animal model tumor systems (Wang and Stoner, 2008), and some pigments can protect lipoproteins and vascular cells from oxidation which is the widely accepted theory for the genesis of atherosclerosis (Wilcox et al., 2003). The importance of pigments has led to the general advice to eat a more varied and colorful diet (WHO-FAO, 2004). Furthermore, anthocyanin accumulation is stimulated by both light intensity (Stutte and Edney, 2009) and quality (Zhang et al., 2018) in red lettuce. As such, Lollo rosso represents a suitable system for cultivation in controlled environment agriculture for studies of both yield and important nutritional quality.

Reports of optimal irradiance for indoor cultivation of lettuce vary between 100 and 600 μmol m^–2^ s^–1^ (Hee and Beom, 2001; Ilieva et al., 2010; Fu et al., 2012; Kang et al., 2013). The ratio of R to B light affects growth in lettuce (Chen et al., 2016; Clavijo-Herrera et al., 2018; Naznin et al., 2019), and there is a clear interaction between the amount of R, B, and W (Son et al., 2016). In addition to the impacts of spectrum, the timing and duration of light treatments may also be significant and although circadian biology is well researched in plant sciences its application to closed environment agriculture is sparse. Many physiological plant processes change over the day in response to environmental signals; others instead follow specific cyclic patterns ascribed as circadian rhythms. Through daily morphological and physiological adaptations, e.g., leaf angle and chloroplast movement, the plant is able to adapt to the fluctuating natural environment by predicting daily changes but also by anticipating regular natural events such as dawn (Dodd et al., 2015). In addition to circadian control, some processes respond cyclically to metabolic feedback; for example, in natural conditions, CO_2_ assimilation usually follows a daily pattern characterized by an initial “photosynthesis activation” at dawn, a maximum CO_2_ fixation at mid-morning, and a decline from midday (midday depression) (Koyama and Takemoto, 2014; Maai et al., 2019). After the midday depression, the photosynthetic activity declines until the dark when the nocturnal process of starch consumption is under circadian oscillator control (Haydon et al., 2013). In controlled environments lacking daylight, complete control of plant rhythms is possible and may be a route to further optimization of light treatments.

In this study, we used a dose-response curve to assess the relationship between irradiance of a broad-spectrum (PAR) LED array and the yield and pigmentation in Lollo rosso. From this curve, we identified a suitable treatment that produced a good combination of yield and morphology that was used to study the effects of PAR plus supplemental R and B on yield and pigmentation. We used supplemental B to examine the effects of duration and to identify a minimal active treatment for further diel studies. We hypothesized that the effectiveness of supplemental B would vary between different diel treatment periods.

## Materials and Methods

### Plant Material and Growth Conditions

Four separate experiments were performed in the same controlled growth room with an 18-h and 6-h light and dark photoperiod. The walls were covered by white reflective sheets (ORCA grow film, California Grow Films LLC), and atmospheric conditions were monitored using Tinytag Ultra 2 (Gemini Data Loggers, Chichester, United Kingdom) and Rotronic CL11 (Rotronic Instruments Ltd., United Kingdom). The average air temperature was 21.9 ± 0.6°C, relative humidity was 58.5 ± 4.8%, and CO_2_ was 470.5 ± 2.4 ppm; averaged environmental values for individual experiments are detailed in Supplementary Table 1.

Seeds of red lettuce Lollo rosso (Antonet RZ seeds from Rijk Zwaan, De Lier, Netherlands) were sown in 155 g of sieved John Innes No. 3 soil-based compost. Water-holding “field” capacity of the compost was calculated following the gravimetric method for soil moisture determination (Reynolds, 1970). Pots were filled, saturated with water, covered with plastic film, and left to drain at room temperature (20 ± 5°C). After 24 h, pot weight was noted and pots were incubated in the oven at 105°C. Every 24 h, pots were weighed until stable dry weight was reached. The dry and wet weights were used to estimate the weight of pots, and soil at approximately 0% and 100% water holding capacity and capacities in between these extremes were estimated as a linear proportion of the difference between these values. Pots were individually irrigated to 80% water holding capacity (205 g) every 48 h until harvest at 30 days after sowing (DAS).

Light intensity and spectral composition of the treatments were measured using the spectroradiometer SpectraPen LM 500 (cosine-corrected, 380–780 nm; Photon Systems Instruments, Drásov, Czechia) (Supplementary Figure 1).

### Broad-Spectrum LED Light Response Curve

Seeds were germinated and grown under two broad-spectrum (PAR) LED arrays (EP006, 380–760 nm, Shenzhen Herifi Technology Co., Ltd., China) (Supplementary Figure 1A) for 30 days. A total of twelve irradiance treatments (5, 10, 15, 30, 40, 60, 80, 100, 120, 140, 160, 180 W m^–2^ or 25, 30, 75, 150, 200, 300, 400, 500, 600, 700, 800, 900 μmol m^–2^ s^–1^) were obtained from the same broad spectrum by adding different layers of muslin cloth as a neutral density filter between source and individual plants. Each treatment was replicated three times and the plants were used for FW determination.

### Supplementing Broad-Spectrum (PAR) LED Arrays With Blue and Red LED Light

Seeds were germinated and grown for 15 days under a broad-spectrum PAR LED source (EP006, 380–760 nm, Shenzhen Herifi Technology Co., Ltd., China) with a photosynthetic photon flux density (PPFD) of 300 μmol m^–2^ s^–1^ (P60). After growth under the P60 array, groups of 9 randomly selected plants were moved under different spectral treatments comprising either double the PAR irradiance (P120), the same PAR irradiance supplemented with R LEDs (P60 + R), or the same PAR irradiance supplemented with B LEDs (P60 + B). Supplemental B and R were provided from mixed arrays of two LEDs centered at 430 and 460 nm (B) and 630 and 660 nm (R) (Supplementary Figures 1B,C). Treatments P120, P60 + R, and P60 + B provided approximately the same PPFD (500 μmol m^–2^ s^–1^). Plants were moved to one of the three supplemental treatments either at 15 DAS or 26 DAS, leaving the remainder under P60 radiation, thus plants were grown under supplemental treatments for either 15 days or 4 days.

### Supplementing Broad-Spectrum (PAR) LEDs With Different Durations of B LEDs

Seeds were germinated and grown for 15 days under a broad-spectrum PAR LED source (EP006, 380–760 nm, Shenzhen Herifi Technology Co., Ltd., China) with a photosynthetic photon flux density (PPFD) of 300 μmol m^–2^ s^–1^ (P60). Plants were randomly selected to either remain under PAR treatment or were grown under similar PAR LEDs supplemented with B radiation to reach a PPFD of 500 μmol m^–2^ s^–1^ (total B accounted for 44% of the emission spectrum). Supplemental B LED treatments varied from a high daily light integral (DLI) B treatment in which plants were transferred to PAR plus supplemental B for the remaining 15 days of the experiment to a minimal DLI B treatment whereby plants remained under P60 for 29 days and received 1 h of PAR60 plus supplemental B LED treatment on the final day (see Table 1 for full range of treatments).

**TABLE 1:** Duration, description, and daily light integrals (DLI) of treatment regimes with PAR plus supplemental B applied for different periods across a 30-days growth period.

**Treatments**	**PAR radiation**	**Supplemental B radiation**	**Total DLI (mol m^–2^ d^–1^)**	**Total B DLI (mol m^–2^ d^–1^)**
P60	30 days (18 h photoperiod)	–	19.44	6.09
B15D	15 days (18 h photoperiod)	15 days (18 h photoperiod)	26.50	14.09
B4D	26 days (18 h photoperiod)	4 days (18 h photoperiod)	21.30	8.22
B2D	28 days (18 h photoperiod)	2 days (18 h photoperiod)	20.38	7.16
B1D	29 days (18 h photoperiod)	1 day (18 h photoperiod)	19.91	6.62
B9h	29 days + 9 h	9 h	19.68	6.36
B4h	29 days + 14 h	4 h	19.54	6.20
B2h	29 days + 16 h	2 h	19.49	6.15
B1h	29 days + 17 h	1 h	19.47	6.12

*Daily light integral (DLI) was calculated by multiplying the instantaneous photon flux density (PFD) (μmol m^–2^ s^–1^) for the total time of the treatment application, then was divided by the number of growth days (30) in order to obtain the mol of photons per day reaching the plant.*

### Interaction of Supplemental B LED Light and the Diel Cycle

**FIGURE 1:**
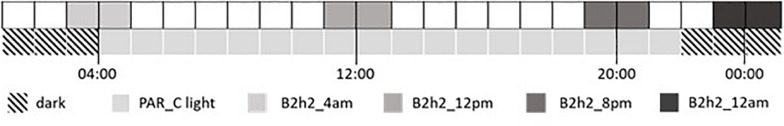
Application times of supplemental B treatment across the diel cycle. The bottom line shows the daily photoperiod of 18 h PAR light (pale gray) and 6 h dark (diagonal stripes). The upper line shows the timing of four supplemental B treatments each applied individually for 2 h.

Seeds were germinated and grown for 15 days under a broad-spectrum PAR LED source (EP006, 380–760 nm, Shenzhen Herifi Technology Co., Ltd., China) with a photosynthetic photon flux density (PPFD) of 300 μmol m^–2^ s^–1^ (P60). Plants were randomly selected to either remain under PAR treatment or were grown for a further 15 days under similar PAR LEDs supplemented with B LED treatments (PPFD of 800 μmol m^–2^ s^–1^) for 2 h during different periods of the day–night cycle. Four treatment times were tested placing the 2-h B treatment at the beginning, middle, and end of the 18-h light period and in the middle of the 6-h dark period (Figure 1).

### Sampling and Measurements of Plant Morphological and Physiological Parameters

Chlorophyll *a* fluorescence was assessed from leaf number four using a portable Handy PEA continuous excitation chlorophyll fluorimeter (Hansatech Instruments Ltd., King’s Lynn, United Kingdom). First, light-adapted measurements were taken, then dark-adapted measurements after 30 min of dark adaptation using the manufacturer’s leaf clips. Measurements were always in the morning just after 10:00, except when treatments required measurements at different specific times.

Rosette images were taken using a fixed focal length digital camera and stand. Images were used for rosette area determination using the “Shape descriptor” plugin in ImageJ software (version 1.52a) (Schneider et al., 2012). The rosette was harvested from just above the cotyledon node and immediately weighed for fresh weight (FW). The entire rosette was then placed in a paper bag and dried to constant weight at 60°C to determine dry weight (DW).

A random selection of plants not used for yield determination was harvested for biochemical analyses at the end of the experiment (day 30). Fully expanded leaves, developmentally the third and fourth leaves, were excised, the midrib was removed, and tissue was immediately frozen in liquid nitrogen before storage at −80°C until analyzed. Prior to analysis, samples were freeze-dried and cold milled to a fine powder in an automated sample grinder (Labman Automation Ltd., Middlesbrough, United Kingdom) for 90 s at −70°C.

### Extraction and Quantification of Anthocyanin Content

Anthocyanins were quantified as a single peak cyanidin 3-malonylglucoside [reported as main anthocyanin in Lollo rosso (Ferreres et al., 1997)] confirmed by fragmentation pattern and mass spectroscopy. Lyophilized powdered leaf material (30 mg) was extracted by shaking in acidified methanol solution (methanol: water: acetic acid; 70: 28.5: 1.5) for 30 min at room temperature. The sample was then centrifuged for 10 min at 1500 × *g*, and the extract was collected and evaporated in a centrifugal evaporator (Jouan, RC 10.22). The concentrated extract was then purified by Solid Phase Extraction using sep-pak cartridges (500 mg Sep-Pak C_18_ 3 cc Vac RC cartridge, Waters Ltd., Elstree, United Kingdom). The final extract was analyzed by reversed-phase HPLC using a Waters system equipped with a 996 photodiode detector array (PDA) and a Nova-Pak C_18_ radial compression column (8 mm × 100 mm, particle size 4 μm; Waters Ltd., Elstree, United Kingdom). The column was equilibrated with 20% solvent A (5% acetic acid) at a flow rate of 2 ml min^–1^. Compounds were eluted by linear gradient to 60% solvent B (100% methanol) over 20 min and monitored from 240 to 600 nm with the detection wavelength set to 525 nm. Anthocyanins were quantified from peak areas using an external standard curve using a cyanidin chloride standard (Sigma-Aldrich Company Ltd.) which had a very similar retention time.

### Statistical Analysis

All the data were statistically analyzed using Microsoft Excel 2016 and R studio [R version 3.5.2 (2018-12-20), “Eggshell Igloo”] with packages agricolae, car, ggplot2, and segmented (Muggeo, 2003; De Mendiburu, 2020; Fox et al., 2020; Wickham et al., 2020). For the measured parameters, data were analyzed by one-way ANOVA and the means were compared by least significance difference (LSD), at 5% significance level.

## Results

### Yield and Morphological Responses to Varying Irradiance of Broad-Spectrum (PAR) LEDs

**FIGURE 2:**
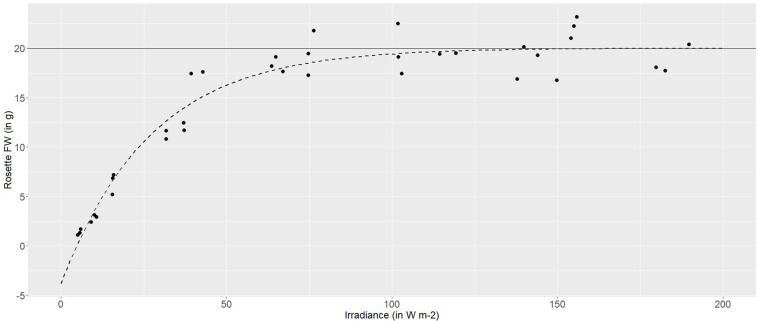
Non-linear (logarithmic) asymptotic regression between the fresh weight (g) of Lollo rosso and irradiance (W m^– 2^). In dark gray is the horizontal asymptote, and in dashed dark gray is the model-based regression curve.

Using a broad-spectrum (PAR) LED array, to provide a range of irradiances between 5 and 180 W m^–2^, demonstrated a highly plastic response in the pigmented lettuce Lollo rosso. Rosette fresh weight (FW) increased logarithmically with increasing irradiance. At lower irradiances, between 5 and 40 W m^–2^, rosette FW increased linearly with increasing irradiance at an average rate of 0.4 g FW per W m^–2^. Higher irradiances, between 40 and 80 W m^–2^, produced 0.1 g per W m^–2^, while irradiances of 100–120 W m^–2^ produced a maximum rosette FW of 20 g and growth was asymptotic at higher irradiances (Figure 2).

Increasing irradiance induced significant changes to morphological responses such as rosette area (*P* = 4.7 × 10^–8^), leaf number (*P* = 2.9 × 10^–9^), and leaf pigmentation (*P* = 4.5 × 10^–5^). Rosette area almost tripled from 5 (73.1 ± 12.7 cm^2^) to 15 W m^–2^ (211.7 ± 4.5 cm^2^), and plants growing under the lower irradiances displayed a prostrate leaf morphology. Rosette area decreased linearly between light energy of 60 and 180 W m^–2^ to a rosette area of 141.8 ± 11.1 cm^2^ (Table 2). Anthocyanins were difficult to detect in plants grown under very low irradiance (up to 15 W m^–2^, 0.18–0.28 mg g^–1^ DW). Light levels of 30 W m^–2^ produced increased accumulation of anthocyanin content (2.07 ± 0.14 mg g^–1^ DW) and reached a maximum under 160 W m^–2^ (6.87 ± 0.63 mg g^–1^ DW) (Table 2).

**TABLE 2:** Morphological responses of Lollo rosso to varying the incident irradiance of a broad-spectrum (PAR) LED array.

**Averaged light treatments (W m^–2^)**	**Averaged light treatment (μ mol m^–2^ s^–1^)**	Leaf number***	Rosette area*** (cm^2^)	Anthocyanin*** (mgCYA g^–1^ DW)
5.5	27.0	5.0 ± 0.0^*f*^	73.1 ± 12.7^*f*^	0.19 ± 0.01^*f*^
9.9	48.2	6.3 ± 0.3^*ef*^	131.2 ± 14.0^*e*^	0.28 ± 0.01^*f*^
15.6	75.4	6.7 ± 0.3^*def*^	211.7 ± 4.5^*a*^	0.18 ± 0.02^*f*^
33.5	161.6	7.7 ± 0.3^*de*^	171.3 ± 7.1^*cd*^	2.07 ± 0.14^*e*^
39.7	186.9	8.7 ± 0.3^*cd*^	200.1 ± 6.2^*abc*^	4.21 ± 0.13^*d*^
65.1	329.7	10.7 ± 1.2^*bc*^	203.4 ± 5.5^*ab*^	5.46 ± 0.08^*c*^
75.3	387.1	13.0 ± 0.6^*a*^	186.6 ± 9.3^*abcd*^	5.72 ± 0.09^*bc*^
102.2	387.1	12.7 ± 0.9^*ab*^	183.1 ± 10.7^*abcd*^	5.75 ± 0.09^*bc*^
125.9	517.9	12.7 ± 1.5^*ab*^	194.9 ± 16.8^*abc*^	6.03 ± 0.53^*abc*^
142.4	731.1	11.7 ± 0.3^*ab*^	157.2 ± 5.8^*de*^	6.55 ± 0.22^*ab*^
154.9	800.0	13.0 ± 0.6^*a*^	180.8 ± 6.6^*bcd*^	6.87 ± 0.63^*a*^
184.0	942.4	12.7 ± 0.3^*ab*^	141.8 ± 11.1^*e*^	6.21 ± 0.74^*abc*^

Values are reported as mean ± standard error of the mean. Different letters within columns indicate significant treatment differences at *P* < 0.05, as determined by analysis of variance (ANOVA) and Fisher’s least significant difference (LSD) test. Significance codes: 0.000 “***”; *n* = 3.

### Supplementing Broad-Spectrum (PAR) LED Arrays With Blue and Red LED Light

There was no significant effect on rosette FW and DW of supplementing broad-spectrum (PAR) LED arrays with R and B LEDs at the irradiances tested (Table 3), but morphology was affected especially rosette area and leaf pigmentation. Rosette area was reduced by the longer (15 days) supplemental treatments (*P* = 0.001). A 15-days supplementation with B LEDs produced the most compact rosette with the lowest area (167.39 ± 3.13 cm^2^), next lowest was 15-days supplementation with PAR LEDs (188.62 ± 6.09), and 15-days supplementation with R LEDs produced a larger rosette area (193.43 ± 4.63). The largest rosette area was produced by Lollo rosso growing under the 4-days supplementation with R LEDs. Accumulation of anthocyanins was enhanced under supplemental B only and was greater in leaves exposed to 4 days than 15 days of supplemental B LEDs (13.00 ± 0.44 and 9.60 ± 0.65 mg g^–1^ DW after 4 days and 15-days B supplementation, respectively) (Table 3) (*P* = 2.2 × 10^–16^).

**TABLE 3:** Fresh and dry weight yield, rosette area, and anthocyanin content of leaf tissue of Lollo rosso lettuce growing under 300 μmol m^–2^ s^–1^ broad-spectrum (PAR) LED (P60) for 15 days and followed by a further 15 days under P60 or P60 plus supplemental PAR, Red (R) or Blue (B) LEDs for either 15 days or 4 days.

**Supplemental treatments**	Rosette area** (cm^2^) (*n* = 3)	**Fresh weight (g) (*n* = 9)**	**Dry weight (g) (*n* = 6)**	Anthocyanin*** (mgCYA g^–1^ DW) (*n* = 3)
–	239.9 ± 14.4^*a*^	20.4 ± 1.0	1.46 ± 0.09	4.96 ± 0.20^*c*^
15D P60	188.6 ± 6.1^*bc*^	17.9 ± 1.2	1.26 ± 0.10	4.14 ± 0.16^*cd*^
4D P60	240.6 ± 11.3^*a*^	19.9 ± 0.7	1.47 ± 0.05	4.42 ± 0.15^*cd*^
15D R	193.4 ± 4.6^*bc*^	21.7 ± 1.1	1.50 ± 0.18	3.77 ± 0.40^*d*^
4D R	246.3 ± 21.4^*a*^	19.8 ± 1.6	1.45 ± 0.09	3.75 ± 0.49^*d*^
15D B	167.4 ± 3.1^*c*^	20.3 ± 0.5	1.44 ± 0.04	9.60 ± 0.65^*b*^
4D B	221.1 ± 7.0^*ab*^	19.8 ± 0.7	1.67 ± 0.08	13.00 ± 0.44^*a*^

Values are reported as mean ± standard error of the mean. Different letters within columns indicate significant treatment differences at *P* < 0.05, as determined by analysis of variance (ANOVA) and Fisher’s least significant difference (LSD) test. Significance codes: 0.000 “***,” 0.001 “**”.

### Supplementing Broad-Spectrum (PAR) LEDs With Different Durations of B LEDs

The duration of supplemental B radiation had a significant effect on rosette FW (*P* = 1.9 × 10^–14^) and DW (*P* = 7.2 × 10^–8^) of Lollo rosso (Table 4). Longer durations of supplemental B increased biomass accumulation which was greatest and asymptotic under 1, 2, 4, and 15 days of supplemental B (19.1 to 20.4 g). Rosette DW followed a similar trend to FW, but the greatest DW was measured after 1 day of supplemental B. Rosette area in general decreased with increasing duration of supplemental B. The smallest rosette areas (1.8-fold smaller than rosettes from control plants) were measured in plants treated with the longest duration of supplemental B (15 days, 146.8 ± 8.5 cm^2^), and the largest rosette area was measured in plants treated with 1 day of supplemental B (181.3 ± 11.5 cm^2^); rosette areas from all the intermediate treatments did not significantly differ (Table 4).

**TABLE 4:** Fresh and dry weight yield, rosette area, and anthocyanin content of leaf tissue of Lollo rosso lettuce growing under 300 μmol m^–2^ s^–1^ broad-spectrum (PAR) LED (P60) for 15 days and then a further 15 days under P60 with different durations of supplemental Blue (B) LEDs.

**Treatments**	Rosette area* (cm^2^) (*N* = 3)	Fresh weight*** (g) (*N* = 8)	Dry weight*** (g) (*N* = 4)	Anthocyanin*** (mgCYA g^–1^ DW) (*N* = 3)
P60	202.9 ± 19.4^*a*^	11.8 ± 0.5^*c*^	0.85 ± 0.13^*d*^	3.98 ± 0.21^*g*^
B15D	146.8 ± 8.5^*c*^	20.4 ± 0.5^*a*^	1.46 ± 0.07^*bc*^	11.44 ± 0.39^*b*^
B4D	174.9 ± 1.5^*b*^	20.2 ± 0.7^*a*^	1.60 ± 0.08^*abc*^	12.91 ± 0.43^*a*^
B2D	169.7 ± 5.1^*bc*^	20.3 ± 1.3^*a*^	1.72 ± 0.10^*ab*^	8.48 ± 0.48^*c*^
B1D	181.3 ± 11.5^*ab*^	19.1 ± 1.6^*a*^	1.76 ± 0.21^*a*^	7.53 ± 0.29^*d*^
B9h	166.2 ± 2.7^*bc*^	15.4 ± 0.8^*b*^	1.44 ± 0.06^*bc*^	7.09 ± 0.09^*de*^
B4h	168.8 ± 4.3^*bc*^	15.7 ± 0.7^*b*^	1.35 ± 0.06^*c*^	6.44 ± 0.05^*e*^
B2h	161.4 ± 3.7^*bc*^	11.2 ± 0.4^*c*^	0.79 ± 0.03^*d*^	5.04 ± 0.15^*f*^
B1h	170.0 ± 4.0^*bc*^	12.0 ± 0.6^*c*^	0.85 ± 0.06^*d*^	3.69 ± 0.04^*g*^

Values are reported as mean ± standard error of the mean. Different letters within columns indicate significant treatment differences at *P* < 0.05, as determined by analysis of variance (ANOVA) and Fisher’s least significant difference (LSD) test. Significance codes: 0.000 “***,” 0.01 “*”.

Supplemental B did not have a significant effect on chlorophyll *a* fluorescence and the maximum quantum efficiency of PSII photochemistry in the dark (F_*V*_/F_*M*_) approximated to a similar value of 0.83 in all measured plants. The amount of light energy dissipated via non-photochemical quenching generally increased with duration of supplemental B, but the differences were not statistically significant.

All supplemental B treatments greater than 1-h duration resulted in significant increases in anthocyanin content compared to levels in leaves from control plants lacking supplemental B (*P* = 3.6 × 10^–14^). The anthocyanin content increased approximately linearly with increasing durations of supplemental B from 2 h (27%) to 4 days (224%) (Table 4). Anthocyanin accumulation declined slightly in leaves exposed to the longest, 15-days, supplemental B treatment (187%). The amount of anthocyanin in leaves exposed to the shortest supplemental B treatment (1 h) did not statistically differ from that in control leaves.

### Interaction of Supplemental B LED Light and the Diel Cycle

When Lollo rosso was grown in an 18-h light, 6-h dark cycle, the timing of a 2-h supplemental B treatment within the light and dark periods had a significant effect on rosette FW (*P* = 0.008). When supplemental B was applied in the middle of the dark period (12 am), rosette FW was greatest. When supplemental B was applied at the end of the light cycle (8 pm), rosette FW was lowest and did not significantly differ from control plants lacking supplemental B. Similar trends were seen in rosette DW (*P* = 0.05) (Table 5).

**TABLE 5:** Fresh and dry weight yield, and anthocyanin content of leaf tissue of Lollo rosso lettuce growing under 300 μmol m^–2^ s^–1^ broad-spectrum (PAR) LED (P60) for 15 days and then a further 15 days under P60 and P60 supplemented with Blue (B) LEDs for 2 h at four different periods.

**Treatments**	Fresh weight** (g) (*n* = 8)	**Dry weight⋅ (g) (*n* = 4)**	Anthocyanin*** (mgCYA g^–1^ DW) (*n* = 4)
P60	10.6 ± 0.6^*b*^	0.79 ± 0.09^*ab*^	4.00 ± 0.14^*c*^
P60 + 2hB (12 am)	12.4 ± 0.4^*a*^	0.92 ± 0.04^*a*^	9.81 ± 0.32^*a*^
P60 + 2hB (4 am)	10.1 ± 0.7^*b*^	0.73 ± 0.06^*b*^	6.60 ± 0.29^*b*^
P60 + 2hB (12 pm)	11.0 ± 0.4^*ab*^	0.75 ± 0.04^*ab*^	7.50 ± 0.48^*b*^
P60 + 2hB (8 pm)	9.6 ± 0.6^*b*^	0.71 ± 0.03^*b*^	4.43 ± 0.44^*c*^

Values are reported as mean ± standard error of the mean. Different letters within columns indicate significant treatment differences at *P* < 0.05, as determined by analysis of variance (ANOVA) and Fisher’s least significant difference (LSD) test. Significance codes: 0.000 “***,” 0.001 “***,” and 0.05 “⋅”.

Anthocyanin content was significantly higher when supplemental B was supplied in the middle of the dark period (9.81 ± 0.37 mg g^–1^ DW) than all other treatment and control plants (*P* = 3.7 × 10^–8^) (Table 5). Supplementing with 2-h B at the beginning (4 am) and middle (12 pm) of the light cycle (6.60 ± 0.29 mg g^–1^ DW and 7.50 ± 0.48 mg g^–1^ DW, respectively) resulted in significantly higher anthocyanin accumulation than control plants (4.00 ± 0.14 mg g^–1^ DW), but anthocyanin contents of control plants and plants treated with supplemental B at the end of the light cycle were not significantly different.

F_*V*_/F_*M*_ was similar between differently treated plants and approximated the optimal value. Maximum operating efficiency of PSII photochemistry in the light (F_*V*_/F_*M*_’) was lowest under midday supplementation and highest under midnight supplementation (*P* = 0.010). Performance index (PI) was similar across the diverse treatments, except the significantly lower values measured when supplementation was applied at the end of the light cycle (*P* ≥ 0.000). Non-photochemical quenching (NPQ) was low and similar in leaves grown in control (P60) and midnight supplemental B treatments but was significantly higher in all three treatments when B was supplemented during the light cycle (*P* = 0.015) (Table 6).

**TABLE 6:** Chlorophyll fluorescence parameters of leaf tissue of Lollo rosso lettuce growing under 300 μmol m^–2^ s^–1^ broad-spectrum (PAR) LED (P60) for 15 days and then a further 15 days under P60 and P60 supplemented with Blue (B) LEDs for 2 h at four different periods.

**Treatments**	F_*V*_/F_*M*_’*	**F_*V*_/F_*M*_**	PI**	NPQ*
P60	0.77 ± 0.01^*ab*^	0.85 ± 0.01	3.01 ± 0.30^*a*^	0.28 ± 0.02^*bc*^
P60 + 2h B (12 am)	0.77 ± 0.01^*a*^	0.85 ± 0.00	3.70 ± 0.22^*a*^	0.23 ± 0.05^*c*^
P60 + 2h B (4 am)	0.75 ± 0.01^*bc*^	0.84 ± 0.00	3.08 ± 0.43^*a*^	0.40 ± 0.04^*ab*^
P60 + 2h B (12 pm)	0.74 ± 0.01^*c*^	0.84 ± 0.01	3.77 ± 0.38^*a*^	0.50 ± 0.06^*a*^
P60 + 2h B (8 pm)	0.74 ± 0.02^*bc*^	0.84 ± 0.01	1.74 ± 0.11^*b*^	0.43 ± 0.08^*ab*^

Values are reported as mean ± standard error of the mean. Different letters within columns indicate significant treatment differences at *P* < 0.05, as determined by analysis of variance (ANOVA) and Fisher’s least significant difference (LSD) test. Significance codes: 0.001 “**,” 0.01 “*”; *n* = 4.

## Discussion

The use of LED lighting is revolutionizing the provision of light to controlled environment agriculture (Pattison et al., 2018). Light intensity has a significant effect on plant growth and morphology (Poorter et al., 2019), and to fully exploit the flexibility afforded by new LED-based lighting systems will involve investigation of the interactions between crop production and variation in irradiance and spectrum (Gómez and Izzo, 2018). Less is known about the potential for further optimization through interactions between light and other factors such as temperature (Franklin et al., 2014) and circadian rhythms (Belbin et al., 2017).

Here we used Lollo rosso, a fast-growing commercial lettuce variety, as a model system to study how irradiance, spectrum, duration, and rhythm of LED light may be used to affect crop growth. We studied aspects of both yield and quality, the latter through the accumulation of anthocyanin pigment. By growing Lollo rosso under a range of PAR irradiance levels, limiting, optimal, and saturating light levels were identified. Light intensity of over 100 W m^–2^ (PAR ∼ 520 μmol m^–2^ s^–1^) produced asymptotic growth, suggesting that photosynthesis and or partitioning of photosynthate into harvestable yield is saturated at such high irradiances. Light exceeding the limiting light levels is more likely to activate photoprotective responses (Robinson, 2001), and light not directly converted to harvestable yield may impact crop quality through alterations in morphology and composition. Rosette area, for instance, decreased in response to irradiance. Under low irradiance light conditions (5–15 W m^–2^), a pale loose-leaf head developed which lacked measurable levels of anthocyanins; such phenotypes are associated with shade conditions to optimize light capture (Björkman and Demmig-Adams, 1995). A more compact rosette head formed and anthocyanin content doubled at moderate light levels (30–40 W m^–2^). At higher irradiances, the rosette area decreased, resulting in more compact rosettes, presumably as a result of hypocotyl length and leaf angle reduction, both traits reported to be protective strategies that decrease incident light interception (Rama Das, 2006; Arsovski et al., 2012). Combining assessments of yield and morphology we concluded that PAR LEDs at 60 W m^–2^ (approximately 300 μmol m^–2^ s^–1^) produced a good combination of yield and morphology in Lollo rosso, and this treatment was chosen to examine supplementing broad spectrum LED light with additional narrow-band LEDs.

Maximal leaf absorbance occurs in the B and R wavelengths due to the absorption peaks of chlorophylls a and b (428–453 and 642–661 nm) and carotenoids (400–500 nm) (McCree, 1971; Barber, 2009). Hence, the previously identified PAR LED irradiance level was supplemented with R or B radiation to reach a higher, but not saturating, light intensity (approximately 500 μmol m^–2^ s^–1^). Our results demonstrated that supplementation of a PAR LED array with additional R or B LEDs had no significant effect on Lollo rosso biomass accumulation. However, supplemental B was effective at stimulating other desirable plant traits including rosette area and anthocyanin content. The reduced rosette area under supplemental B probably resulted from previously demonstrated effects of B inhibiting stem elongation and controlling leaf orientation (Huché-thélier et al., 2016). The increase in cyanidin content compared to PAR treatments lacking supplemental B was greater in the shorter B treatment. In contrast, NPQ increased with duration of supplemental B and a similar contrast was reported in young leaves of *Acmena acuminatissima* in contrasting seasons by Zhu et al. (2018). The phenotype of decreasing pigmentation and increasing stress may be due to alternative acclimation responses (Nogués et al., 1998), with the plant investing more in internal and constitutive protective mechanisms such as energy dissipation as heat through the xanthophyll cycle or rearrangements of photosystem machinery, rather than adopting largely preventive strategies through increased pigmentation (Steyn et al., 2002; Bailey et al., 2004; Li et al., 2009).

Having established the potential for shorter-duration supplemental B to improve pigmentation and morphology, we next determined the relationship between duration of supplemental B treatment and pigmentation to identify a minimal treatment condition. Identifying a short enough treatment would enable us to further investigate the interaction of supplementation and the diel growth cycle. If absorbed by plants, the highly energetic photons of short wavelength light may increase photoprotection responses and negatively impact plant growth (Landi et al., 2015). UV radiation, for example, has been widely studied in this respect and stimulates the accumulation of secondary metabolites in plants (Schreiner et al., 2012) and inhibits growth in many plant species including lettuce (Tsormpatsidis et al., 2010; Lee et al., 2013). In contrast to UV, B wavelengths are photosynthetically active (Zhu et al., 2018), B LEDs are readily available commercially and can produce light at a higher efficiency (93%) compared with 81% for R LEDs (Kusuma et al., 2020). Thus, supplementation with B LEDs represents a suitable system to examine ways to increase photoassimilation and increase pigmentation while minimizing the negative impacts of phototoxicity, non-photochemical quenching, and consequent reduction in yield.

Exposing Lollo rosso to supplemental B for decreasing periods of time (from 15 days to 1 h) produced a distinct dose–response effect on the measured variables. Leaf anthocyanin content increased linearly from 2 h to 4 days of B supplementation. Rosette area and NPQ increased proportionally with treatment duration. The plateau in yield at around 1 day of supplemental B treatment suggests that the extra B photons were no longer contributing to net biomass accumulation at longer durations and the decrease in leaf anthocyanin content after the longest B treatment may be caused by acclimation of the plant to the high light environment. A similar response was reported by Taulavuoria et al. (2016) in which many compounds in basil decreased after B treatment for 48 days compared to a shorter 36-days treatment.

A minimal treatment of 2-h supplemental B was sufficient to significantly increase cyanidin content but had no significant effect on biomass. Thus, in the next test, the 2-h B treatment was adjusted to deliver an equivalent DLI to the most effective 4 days treatment (∼21.3 mol m^–2^ d^–1^, Table 4) and broad-spectrum PAR LEDs were supplemented with the resulting 2-h B at different times in the diel cycle. Increasing daily light integral (DLI), or the total sum of radiation in a 24-h period, allows higher radiation sums with lower PPFD, thus avoiding negative effects of saturating light levels and increasing lettuce fresh and dry mass (Kelly et al., 2020). Supplemental B at midday resulted in the highest NPQ which could be associated with midday depression of photosynthesis, stomatal closure, and reduced ability to dissipate light energy photochemically (Koyama and Takemoto, 2014). The lower total PPFD in the night break treatment (545 compared to 830 μmol m^–2^ s^–1^) may allow most incident B photons to be photochemically quenched explaining the high and unaffected F_*V*_/F_*M*_’ and the low level of NPQ. B light application in the night period was reported to increase carbon export and enhance fruit production (Lanoue et al., 2019), and the night break produced the highest biomass of our diel treatments. The higher percentage of B in the total PPF, 100% in the midnight treatment compared to 75% in PAR LEDs supplemented with B radiation, may explain the greater effectiveness at inducing anthocyanins of the supplemental B night break since anthocyanin content has been reported to increase with the percentage of B (Hernández et al., 2016).

Our data demonstrates the effectivity of supplemental B LEDs in stimulating leaf cyanidin accumulation while enhancing plant growth in Lollo rosso. Our results suggest that application of short-duration supplemental B LED light to PAR arrays has beneficial effects on Lollo rosso yield, morphology, and pigmentation and further that shorter duration supplementation may be made even more effective if applied during night breaks. Such studies will help producers improve crop quality and maximize returns on energy input from supplemental LED lighting utilizing visible wavelengths.

## Data Availability Statement

The original contributions presented in the study are included in the article/Supplementary Material, further inquiries can be directed to the corresponding author.

## Author Contributions

LC: conceptualization, methodology, investigation, formal analysis, writing – original draft, and writing – reviewing and editing. ID: funding acquisition and supervision. PR: funding acquisition, conceptualization, resources, writing – original draft, writing – reviewing and editing, and supervision. All authors contributed to the article and approved the submitted version.

## Conflict of Interest

The authors declare that the research was conducted in the absence of any commercial or financial relationships that could be construed as a potential conflict of interest.

## Supplementary Material

Supplementary Figure 1 Light spectral composition of the PAR array (**A**, solid line in pink), the blue LEDs (**B**, dashed line in blue), and the red LEDs (**C**, dotted line in red). Each line represents the average of three measurements recorded at different spots of the plant canopy.

Supplementary Table 1 Environmental data for the experiments reported. Values are reported as mean ± standard error of the mean.

Supplementary Table 2 Maximum quantum efficiency of PSII (F_*V*_/F_*M*_) and non-photochemical quenching (NPQ) of Lollo rosso leaf growing under 60 W m^–2^ broad spectrum (PAR) LED (P60) for 15 days and followed by a further 15 days under P60 or P60 plus supplemental PAR, Red (R) or Blue (B) LEDs for either 15 days or 4 days. Values are reported as mean ± standard error of the mean.
