# Supplementary figures and images for: Producing Enhanced Yield and Nutritional Pigmentation in Lollo Rosso Through Manipulating the Irradiance, Duration, and Periodicity of LEDs in the Visible Region of Light
Source: Front Plant Sci. 2020 Dec 18;11:598082. doi: 10.3389/fpls.2020.598082 (PMC7775386; doi:10.3389/fpls.2020.598082)

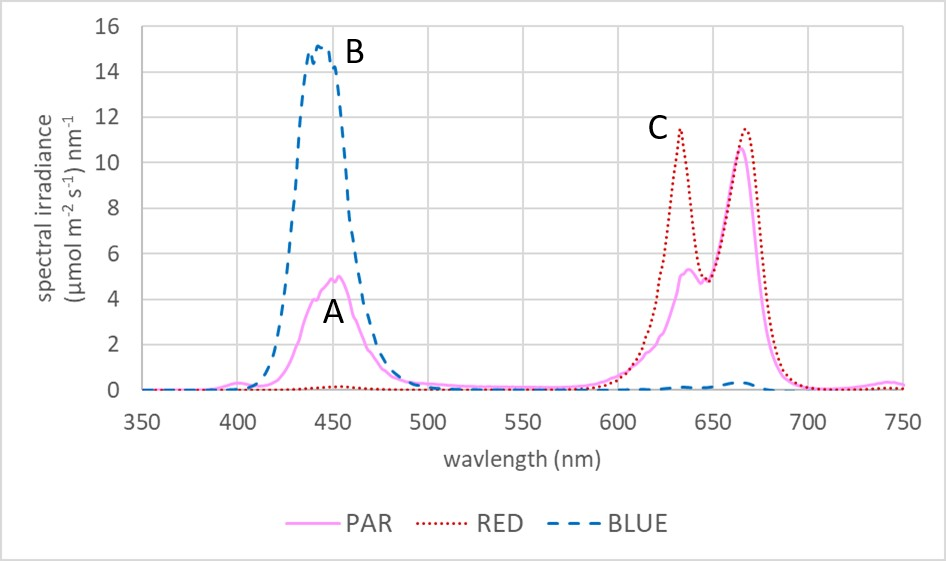

Supplement: Supplementary Figure 1 — Light spectral composition of the PAR array (A, solid line in pink), the blue LEDs (B, dashed line in blue), and the red LEDs (C, dotted line in red). Each line represents the average of three measurements recorded at different spots of the plant canopy. [file Image_1.png]
