# Supplementary material for: Producing Enhanced Yield and Nutritional Pigmentation in Lollo Rosso Through Manipulating the Irradiance, Duration, and Periodicity of LEDs in the Visible Region of Light
Source: Front Plant Sci. 2020 Dec 18;11:598082. doi: 10.3389/fpls.2020.598082 (PMC7775386; doi:10.3389/fpls.2020.598082)
Supplement: Supplementary Table 1 — Environmental data for the experiments reported. Values are reported as mean ± standard error of the mean. [file Table_1.docx]

Table S1. Environmental data for the experiments reported.

| Experiment | Temperature (°C) | Relative Humidity (%) | Carbon dioxide (ppm) |
| --- | --- | --- | --- |
| Broad-spectrum LED light response curve | 22.41 ± 0.03 | 55.14 ± 0.29 | 497.34 ± 3.78 |
| Supplementing broad-spectrum (PAR) LED arrays with B and R LED light | 24.10 ± 0.40 | 51.85 ± 2.02 | 466.31 ± 4.83 |
| Supplementing broad spectrum (PAR) LEDs with different durations of B LEDs | 22.59 ± 0.03 | 51.60 ± 0.20 | 464.65 ± 2.34 |
| Interaction of supplemental B LED light and the diel cycle | 20.61 ± 0.03 | 48.77 ± 0.34 | 446.84 ± 1.30 |

Values are reported as mean ± standard error of the mean.
