# Supplementary material for: Producing Enhanced Yield and Nutritional Pigmentation in Lollo Rosso Through Manipulating the Irradiance, Duration, and Periodicity of LEDs in the Visible Region of Light
Source: Front Plant Sci. 2020 Dec 18;11:598082. doi: 10.3389/fpls.2020.598082 (PMC7775386; doi:10.3389/fpls.2020.598082)
Supplement: Supplementary Table 2 — Maximum quantum efficiency of PSII (FV/FM) and non-photochemical quenching (NPQ) of Lollo rosso leaf growing under 60 W m–2 broad spectrum (PAR) LED (P60) for 15 days and followed by a further 15 days under P60 or P60 plus supplemental PAR, Red (R) or Blue (B) LEDs for either 15 days or 4 days. Values are reported as mean ± standard error of the mean. [file Table_2.docx]

Table S2. Maximum quantum efficiency of PSII (F_V_/F_M_) and non-photochemical quenching (NPQ) of Lollo rosso leaf growing under 60 W m^-2^ broad spectrum (PAR) LED (P60) for 15 d and followed by a further 15 d under P60 or P60 plus supplemental PAR, Red (R) or Blue (B) LEDs for either 15 d or 4d.

| Supplemental treatments | F_V_/F_M_  (N=3) | NPQ  (N=3) |
| --- | --- | --- |
| - - | 0.85 ± 0.01 | 0.35 ± 0.09 |
| 15D P60 | 0.84 ± 0.01 | 0.51 ± 0.22 |
| 4D P60 | 0.85 ± 0.01 | 0.43 ± 0.04 |
| 15D R | 0.84 ± 0.00 | 0.79 ± 0.28 |
| 4D R | 0.83 ± 0.01 | 0.58 ± 0.18 |
| 15D B | 0.84 ± 0.01 | 0.60 ± 0.16 |
| 4D B | 0.84 ± 0.01 | 0.45 ± 0.11 |

Values are reported as mean ± standard error of the mean.
